# Antioxidant and anti-inflammatory properties of *Erythroxylum cuneatum* alkaloid leaf extract

**DOI:** 10.1016/j.heliyon.2020.e04141

**Published:** 2020-06-28

**Authors:** Lim Seow Li, Samaila Musa Chiroma, Thuaibah Hashim, Siti Khadijah Adam, Mohamad Aris Mohd Moklas, Zauyah Yusuf, Shamima Abdul Rahman

**Affiliations:** a Department of Pharmaceutical Sciences, Faculty of Pharmacy, University of Cyberjaya, 63000, Cyberjaya, Malaysia; b Department of Pathology, Faculty of Medicine, University of Cyberjaya, 63000, Cyberjaya, Malaysia; c Department of Human and Anatomy, Faculty of Medicines and Health Sciences, Universiti Putra Malaysia, 43000, Selangor, Malaysia; d Department of Human Anatomy, Faculty of Basic Medical Sciences, University of Maiduguri, Maiduguri, 600231, Borno state, Nigeria

**Keywords:** Alkaloid extraction, *Erythroxylum cuneatum*, Antioxidant, Anti-inflammatory, Carrageenan induced edema, Physiology, Anatomy, Pharmacology, Alternative medicine, Evidence-based medicine

## Abstract

*Erythroxylum cuneatum* (*E*. *cuneatum*) which belongs to Erythroxylaceae family is a tropical flowering plant from the genus of Erythroxylum. It is used in Malaysia and Thailand's traditional medicines, yet there is limited scientific reports on its medicinal value. This study aimed at exploring the antioxidative and anti-inflammatory properties of *E. cuneatum* alkaloid leaf extract. The alkaloid extract was obtained through Soxhlet heat extraction method, while the antioxidantive properties were assessed via 2,2-diphenyl-1-picrylhydrazyl (DPPH) free radical scavenging, ferric reducing antioxidant power (FRAP) and xanthine oxidase inhibition (XOI) assays. Further, anti-inflammatory property of the extract was evaluated on rat's model of carrageenan induced paw model of edema via physical measurements and histology. The extract exhibited antioxidant activity with an EC_50_ value of 1482 μg/ml in the DPPH radical scavenging assay, an EC_1_ value of 2191 μg/ml in the FRAP assay and 10.15 ± 6.20% in the XOI assay. Rats pretreated with the extract have shown dose dependent decrease in paw edema when compared to non-treated group of rats. The highest dose (50 mg/kg) of extract exhibited similar effects to aspirin in terms of reducing paw thickness, leucocytes infiltration and disruption of collagen. In conclusion, the *E. cuneatum* alkaloid leaf extract possesses both antioxidative and anti-inflammatory properties suggesting its potentials for future development of antioxidant and anti-inflammatory drugs.

## 1 Introduction

Erythroxylum cuneatum (*E. cuneatum*) is a shrub which is locally known as Cinta mula, ketai mula, inai-inai or baka in Malaysia [1]. The plant was found to be rich in tropane alkaloid as reported earlier and used by the locals in Malaysia on women who had miscarriage [2].

Inflammation is the body response towards foreign substances such as pathogens, allergens, chemical irritants as well as injury which involves infiltration of leukocytes and generation of pro-inflammation factors to the injured site. Inflammation helps in mitigating the effects of harmful microorganisms and remove dead cells which may prevent further development of irritation and allows the injured tissue to recover to normal condition.

Reactive oxygen species (ROS) is the product of cellular aerobic metabolism giving rise to both harmful and beneficial effects to the body. Inequality between antioxidants and oxidants in the body is detrimental to the integrity of macromolecules and body cells [3]. Free radicals generation is one of the causes of inflammation, while excessive and persistent inflammation leads to undesirable pathologic conditions such as rheumatoid arthritis, neurogenerative diseases, cancer, asthma and inflammatory bowel disease [4].

Drugs that are commonly used for the treatments of inflammatory conditions are known as non-steroidal anti-inflammatory drugs (NSAIDs) and corticosteroids. Non-selective NSAIDs are associated with gastric ulcerogenesis which occurs in patients after long term exposure, while selective NSAIDs have displayed better gastrointestinal tolerability and safety. However, toxicities and kidney problems are not uncommon to both the non-selective and selective NSAIDs. Further, selective NSAIDs are also identified for increasing the risk of developing cardiovascular diseases such as myocardial infarction and stroke which led to worldwide withdrawal of rofecoxib [5, 6]. Corticosteroids, like the NSAIDs are also associated with numerous side effects such as gastrointestinal ulcers, hyperglycaemia and steroids withdrawal symptoms [7]. This prompts the concern in searching for natural compounds that could mimic the effects of synthetic NSAIDs but with less side effects. Although *E. cuneatum* are commonly being used as folk medicine for anti-inflammatory and anti-pyretic purposes, there is limited scientific investigation of these properties. Hence this study has been undertaken to evaluate the antioxidative and anti-inflammatory properties of *E. cuneatum* alkaloid leaf extract.

## 2 Materials and methods

### 2.1 Chemicals

The chemicals used for this study included, methanol, glacial acetic acid, ammonia, ascorbic acid and chloroform all purchased from HmbG Chemical, Malaysia. Others includes, n-hexane and ferric chloride hexahydrate (FeCl_3_.6H_2_O) purchased from Bendosen, Malaysia. 2-diphenyl-1-picrylhydrazyl (DPPH) was procured from Sigma, Switzerland while sodium acetate, xanthine, xanthine oxidase and allopurinol were purchased from Sigma-Aldrich, Germany. Additionally, sodium dihydogen phosphate (NaH_2_PO_4_), disodium hydrogen phosphate (Na_2_HPO_4_), carrageenan, Tween 20, aspirin, sodium chloride, Dragendorff's and Mayer's reagent were purchased from R&M chemical, Malaysia. Haematoxylin, putts eosin and DPX were purchased from Cellpath, UK, hydrochloric acid (HCl), formaldehyde, xyleneand ethanol were purchased from Systerm, UN. Finally, 2,4,6-tri(2-pyridyl)-s-triazine (TPTZ) (Acros, US), ketamine (Ketaset®), xylazine 100 mg/ml (Rompun®), ferrous sulphate heptahydrate (FeSO4.7H_2_O) (Fisher chemical, US) and paraffin wax (LEICA, Germany) were purchased from the respective sources.

### 2.2 Sources of *E. cuneatum*

The identified plant material was confirmed and validated by a botanist from Institute of Bioscience, Universiti Putra Malaysia (UPM), Serdang, Selangor. The voucher (SK 2100/12) has been deposited at the herbarium in UPM for future reference, while three kilograms of the leaf powder of *E. cuneatum* was used for the purpose of this study.

### 2.3 Preparation of *E. cuneatum* alkaloid leaf extract

The alkaloid extraction was done with Soxhlet heat extraction method. The boiling flask was filled with methanol with the extraction fraction ratio of 1:7 (leaf powder: solvent). The Soxhlet heat extraction was conducted until the siphon tube changed its color from green to colorless. Then, the methanol extract was concentrated using BUCHI rotary evaporator at 40 °C. The concentrated extract was then soaked overnight in 10% of acetic acid and filtered. The collected filtrate was fractionated twice with hexane in ratio of 1:1 using separatory funnel. The fractionation process was achieved when two separated layers are formed. The bottom acetic acid layer was collected and mixed with 25% of ammonia until the mixture reached pH 10. After which, the alkalinized solution was filtered and fractionated twice with chloroform in the ratio of 1:1. The bottom chloroform layer was collected and concentrated with rotary evaporator at 40 °C. The viscous extract formed was then stored at 4 °C for later use. The percentage yield (%) of the extract was calculated using the formula;

Weight of alkaloid residue (g)/ weight of ground leaf powder (g) x 100%.

### 2.4 Confirmation for the presence of alkaloid in *E. cuneatum* leaf extract

The extract was verified for the presence of alkaloid using Mayer's reagent and thin layer chromatography. In Mayer's reagent assay, 3 ml of diluted HCL was added to two flasks, one contained little amount of the extract and the other one without the extract, respectively. Then, several drops of Mayer's reagent were added. Formation of yellow precipitate after 1 min in the flask containing the extract indicates the presence of alkaloid [8]. The extract was then checked in thin layer chromatography(TLC) on analytical plates over silica gel. The plant extracts were applied on pre-coated TLC plates using capillary tubes and developed in a TLC chamber using suitable mobile phase. The developed TLC plates were air dried and observed under ultra-violet light UV at 225 nm. They were later sprayed with Dragendorff spraying reagent and were placed in hot air oven for 1 min for the development of color in separated bands. The presence of alkaloids in extract were based on spots appeared [9].

### 2.5 Antioxidant studies

#### 2.5.1 2,2-diphenyl-1-picrylhydrazyl (DPPH)

The free radicals scavenging activity of *E. cuneatum* alkaloid leaf extract was evaluated using DPPH reagent through a method described by Dickson and Sahgal [10, 11] with little modification. Briefly, the reaction mixture consisted of DPPH reagent and *E. cuneatum* alkaloid leaf extract with different concentrations incubated in a dark environment. The absorbance was read at 517 nm using UV-VIS spectrophotometer and the experiment was performed in triplicate. Ascorbic acid was used as the reference standard while negative control was the reaction mixture with DPPH and methanol only. Finally, the percentage of scavenging effect (%) was calculated using the formula = (1- α/ß) x 100%, whereby α is the absorbance of test extract and ß is the absorbance of negative control. The results were expressed as mean ± SD. EC50 value (mg/ml) was defined as the total antioxidant required to reduce the initial DPPH free radicals by 50%. It was determined from the graph of percentage of scavenging activity plotted against various concentrations of extract and ascorbic acid using non-linear regression dose-response logarithmic function curve using GraphPad Prism 6.0 software.

#### 2.5.2 Ferric reducing antioxidant power assay (FRAP)

FRAP assay was performed as described by Benzi and Strain [12] with slight modifications ([13] Briefly, FRAP reagent was prepared by mixing acetate buffer (200 ml, 300 mM, pH 3.6), 10 mM TPTZ solution (20 ml) in 40 mM HCl, and 20 mM FeCl_3_ solution (20 ml) in proportions of 10:1:1 (v/v), respectively. The FRAP reagent was freshly prepared and warmed to 37 °C prior to use. The reaction mixture consisted of 100 μL of alkaloid extract of *E.* *cuneatum* (1–2000 μg/ml) with 300 μL of 2% Tween 20 and 3 ml of FRAP reagentand incubated for 30 min. Then, the absorbance of reaction mixture was measured at 593 nm. Ascorbic acid (1–500 μg/ml) was used as a comparative model for the reaction mixtures. A standard curve was constructed by using a serial dilution of ferrous sulphate heptahydrate (FeSO4.7H_2_O) at a range between 0.1 to 2.0 mM. The assay was measured in triplicates and expressed as mM Fe(II) per gram of dry weight of plant material. Finally, EC1 was evaluated as the concentration of antioxidant giving an absorbance increase in the FRAP assay equivalent to the theoretical absorbance value of 1 mM concentration of Fe(II) solution determined using the corresponding regression equation.

#### 2.5.3 Xanthine oxidase inhibitory assay

Xanthine oxidase activity was determined by measuring the formation of uric acid from xanthine. The *E. cuneatum* alkaloid leaf extract and allopurinol (reference standard) were dissolved and diluted with 2% Tween 20 to get concentration of 1–10000 μg/ml of test samples. The reaction mixture consisting of 0.1 ml of samples with 1.9 ml of 50 mM phosphate buffer solution (pH 7.5) was pre-incubated at 37 °C for 15 min. The enzymatic reaction was then initiated by adding enzyme xanthine oxidase and the reaction was stopped after 15 mins using 0.5 M HCl. The absorbance of uric acid was measured at 290 nm. The blank used was buffer and the negative control was a solution containing xanthine and xanthine oxidase. The inhibition percentage of xanthine oxidase inhibitory activity (%) was calculated according to the formula = (1- α/ß) x 100%, whereby α is the absorbance of test samples and ß is the absorbance of negative control. The inhibition concentration (IC50) that inhibited 50% of uric acid production by allopurinol and extract was evaluated and compared by constructing a dose-response logarithmic function curve using non-linear regression by GraphPad Prism 6.0 software [14, 15].

### 2.6 Experimental animals

A total of 42 healthy male Sprague-Dawley rats (200–250 g) were used in this study. The rats were kept in polyacrylic cages with not more than four rats per cage and housed under standard laboratory conditions of 12 h light/12 h dark cycle at 22 ± 2 °C with free access to standard rat chow and water. The rats were kept for one week acclimatization before the experiment begun. The experimental protocol used in this study was approved by Institutional Animal Care and Use Committee (IACUC) of UPM with the reference number UPM/IACUC/AUP-R075/2013.

### 2.7 Experimental design for animal study

**Figure 1:**
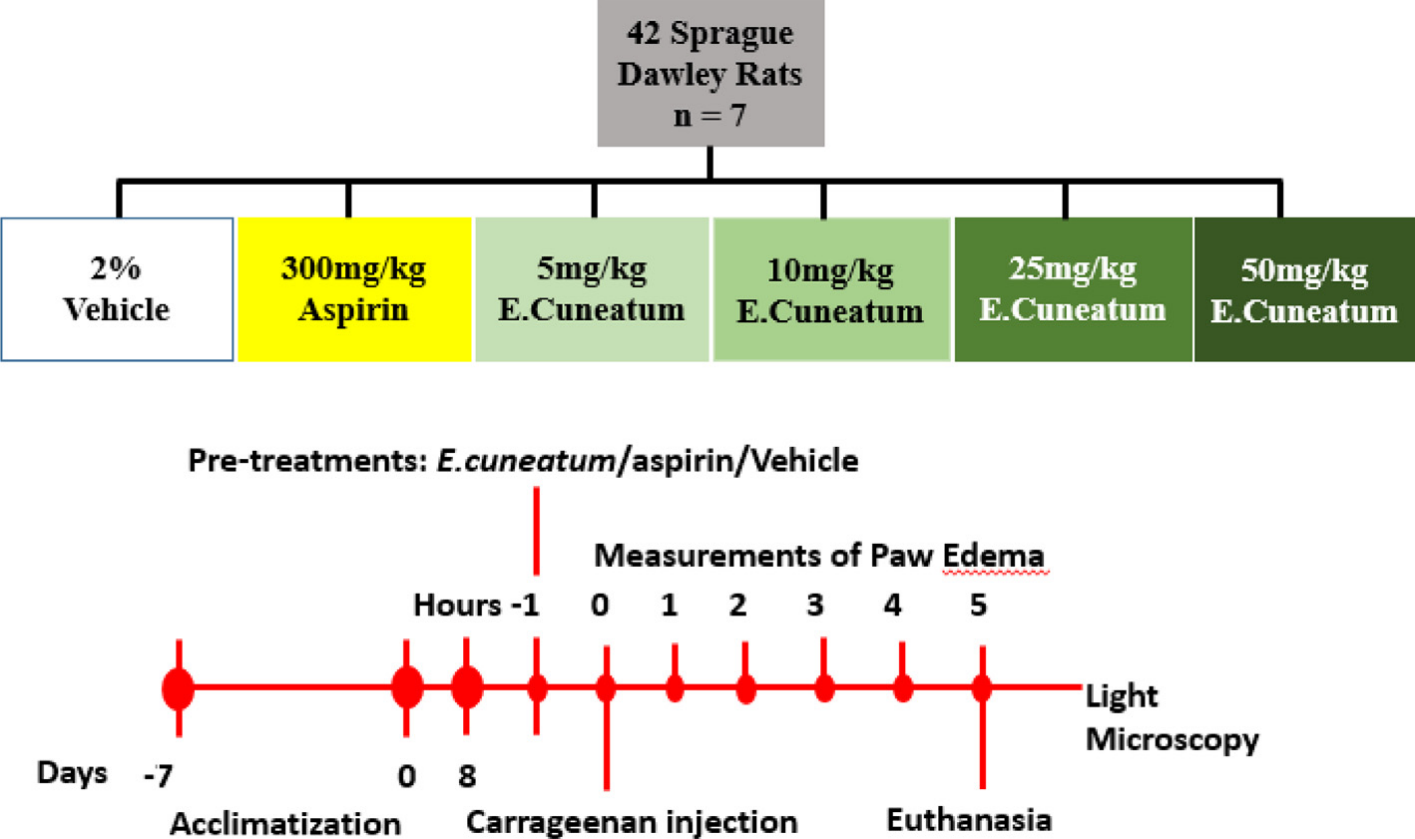
Experimental design for evaluation of anti-inflammatory effects of *E. cuneatum* alkaloid leaf extract on carrageenan induced edema in rats paw.

To determine the anti-inflammatory activity of *E. cuneatum* alkaloid leaf extract, the experimental rats were randomly divided into 6 groups. Group 1 (negative control) received 2% Tween 20; Group 2 (positive control) received 300 mg/kg Aspirin; Group 3, 4, 5 and 6 as the treatment groups received 5 mg/kg, 10 mg/kg, 25 mg/kg, and 50 mg/kg of *E. cuneatum* alkaloid leaf extracts respectively (Figure 1).

### 2.8 Carrageenan-induced paw edema acute inflammatory model

The rats were pre-treated with either aspirin, different doses of alkaloid extract of *E. cuneatum* alkaloid leaf extract or vehicle via oral force feeding one hour prior to subplantar injection of 0.2 ml of 1% (w/v) carrageenan suspension to their right hind paw. The time when the injection was given was recorded as 0 h, while the paw thickness of each rat was measured using vernier caliper just before the injection was carried-out. One hour after induction of the edema, the thickness of the paw of each rat was measured and the time was recorded as first (1st) hour, then the paw's thickness was subsequently measured at hourly basis up to the 5th hour. The increase of paw thickness for every hour was calculated as difference of paw thickness at particular (t) hour to 0 h and presented as mean increase of paw thickness (cm). The anti-inflammatory effect of standard drug and *E. cuneatum* alkaloid leaf extract was expressed as percentage of inhibition (%) which was using the calculated formula = [1- (Ct – C0) treated group/(Ct – C0) control group] x 100%, where C0 is mean paw thickness measured at time 0 h and Ct is the mean paw thickness measured at particular time point [16].

### 2.9 Histology

The rats were euthanized, and their inflamed right hind paws were excised and preserved in 10% of formalin for one week. The tissues were then processed and stained with hematoxylin and eosin (H&E). The stained slides were selected randomly and captured under magnification 200X using digital image analyzer. The scoring the tissues was conducted according to the methods described by Danica [17] with little modifications for grading of the tissues. The scores for inflammation were defined as: 0 = no inflammation, 1 = mild inflammation, 2 = moderate inflammation and 3 = severe inflammation.

### 2.10 Statistical analysis

For antioxidant studies, the results were expressed as mean ± SD and statistical analyses were performed by independent t-test. For animal study, the data were analyzed using one-way analysis of variance (ANOVA), followed by Tukey's post hoc comparison where necessary. Results were expressed as mean ± SEM, SPSS version 20.0 software was used for the statistical analysis.

## 3 Results

### 3.1 Percentage yield of *E. cuneatum* alkaloid leaf extract and the confirmation of presence of alkaloid

**Figure 2:**
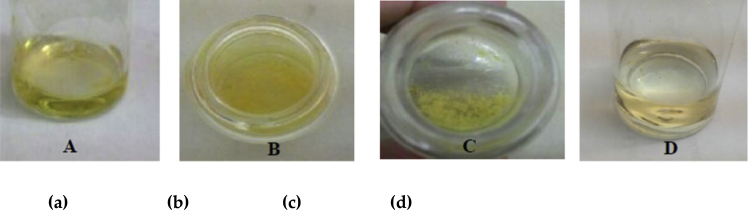
The screening for the presence of alkaloid in *E. cuneatum* leaf extract. The pictures indicate (A) extract + diluted HCl, (B) extract + diluted HCl + Mayer's reagent, (C) precipitation formed 1 min after drops of Mayer's reagent and (D) diluted HCL + Mayer's reagent only.

The percentage yield of alkaloid extraction was 0.12%. The confirmation of the presence of alkaloid in the extract was done using Mayer's reagent. Formation of yellow precipitate confirmed the presence of alkaloid in the extract as shown in Figure 2.

**Figure 3:**
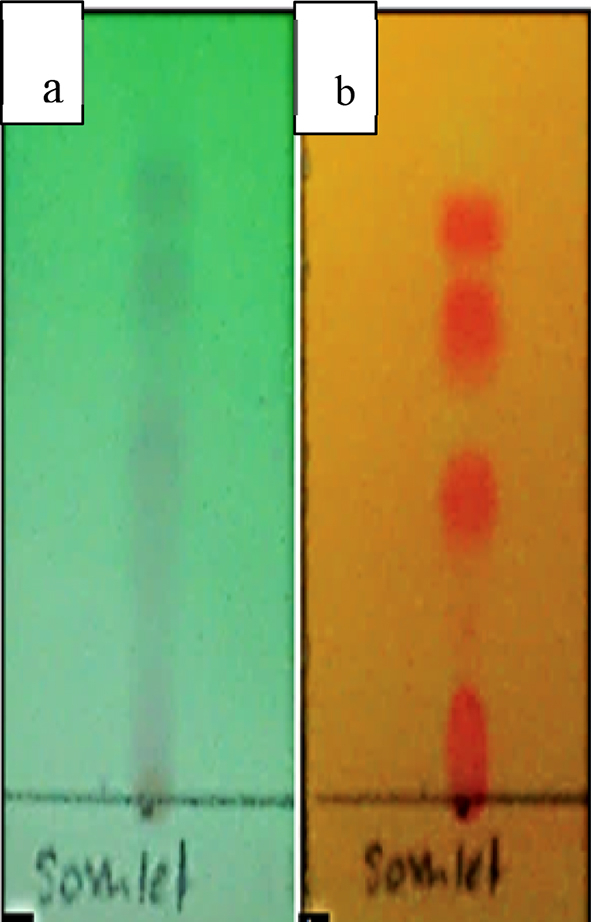
The screening of the presence of alkaloid in *E. cuneatum* leaf extract using TLC. The pictures indicate (a) Under 225 nm ultraviolet light and (b) Stained with Dragendorff ’s reagent.

Further analysis with thin layer chromatography showed four spots appeared on the TLC paper, which were the separation of distinct and separate alkaloid compounds. The best solvent system used for alkaloid extract was chloroform: diethylamine (9:1). Thin layer chromatography spots of alkaloid extract from *E. cuneatum* leaves were shown in Figure 3.

### 3.2 2,2-Diphenyl-1-picrylhydrazyl (DPPH) free radical scavenging assay

**Figure 4:**
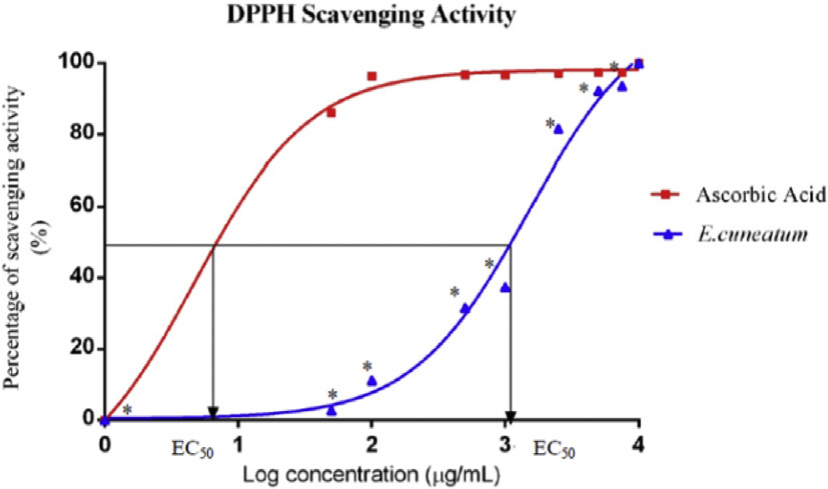
Percentage of free radical scavenging activities of *E. cuneatum alkaloid leaf extract* and ascorbic acid measured at various concentrations and their EC50. Values expressed as mean ± SD with n = 3/concentration and EC50 is defined as half maximal effective concentration. Log concentration of 0, 1, 2, 3, and 4 μg/ml represent concentration of 1, 10, 100, 1000 and 10000 μg/ml, respectively. ∗: is significant when compared to ascorbic acid (p < 0.05) according to independent t-test.

The *E. cuneatum* alkaloid leaf extract and ascorbic acid showed a dose-response relationship with the scavenging activities which were directly proportional to their concentrations, but with a non-linear pattern (Figure 4). The *E. cuneatum* alkaloid leaf extract showed significant lower activities for every concentration when compared to ascorbic acid (p < 0.05), except at the highest concentration. Both extracts showed similar activity at 10,000 μg/ml, which was 100% (p > 0.05). The EC50 value for ascorbic acid was 4.69 μg/ml, which was significantly lower than *E. cuneatum* alkaloid leaf extract (1482 μg/ml).

### 3.3 Ferric reducing antioxidant power assay

**Figure 5:**
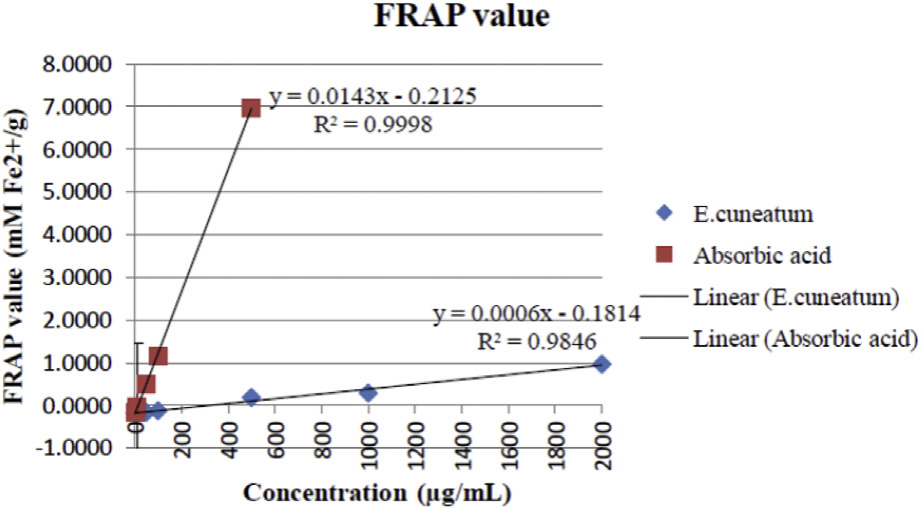
FRAP value of *E. cuneatum* alkaloid leaf extract and ascorbic acid, expressed as mM Fe2+/g of extract. Values expressed as mean ± SD, n = 3/concentration.

FeSO_4_.7H_2_O standard curve constructed with good linearity, y = 0.7278x + 0.0786 (R2 = 0.9886) was used for the determination of antioxidant power of *E. cuneatum* alkaloid leaf extract and ascorbic acid. The FRAP values of *E. cuneatum* alkaloid leaf extract at all concentrations were significantly lower than ascorbic acid as shown in Figure 5. There was no ferric reducing activity for the *E. cuneatum* alkaloid leaf extract with concentrations lower than 300 μg/ml. The activity increased gradually for the subsequent concentrations, with the highest FRAP values of 0.9644 ± 0.00 mM Fe (II)/g at 2000 μg/ml. In contrast, 100 μg/ml of ascorbic acid was able to give FRAP value of 1.1478 ± 0.00 mM Fe(II)/g. The Equivalent Concentration (EC1) was employed for better comparison of activity of *E. cuneatum* alkaloid leaf extract and the ascorbic acid. The EC1 for *E. cuneatum* alkaloid leaf extract and ascorbic acid were 2191 μg/ml and 94.35 μg/ml, respectively.

### 3.4 Xanthine oxidase inhibition assay

**Figure 6:**
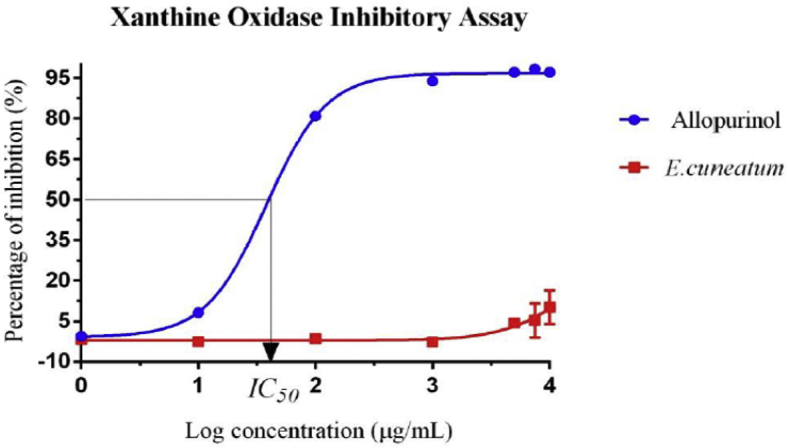
Xanthine oxidase inhibition dose-response effect. Results expressed as mean ± SD, n = 3/concentration. Log concentration of 0, 1, 2, 3, and 4 μg/ml represent concentration of 1, 10, 100, 1000 and 10000 μg/ml, respectively. IC50 is the concentration of allopurinol needed to inhibit 50% of XO inhibitory activity.

Allopurinol demonstrated a dose-response relationship of XO inhibitory activities but *E. cuneatum* alkaloid leaf extract does not show any comparable activity when compared to the standard (Figure 6). The maximum percentage of inhibition of allopurinol was 98.34 ± 0.09% at the concentration of 7500 μg/ml and the minimum activity was 8.24 ± 0.93% at the concentration of 10 μg/ml. While the *E. cuneatum* alkaloid leaf extract only showed positive activity when it reached 5000 μg/ml and maximum XO inhibition activity of the *E. cuneatum* alkaloid leaf extract was only 10.15 ± 6.20% (10000 μg/ml). The IC50 of allopurinol was 38.81 μg/ml from the equation y = -0.8721 + 97.6421/[1 + 10(1.581-x) (1.689)], (R2 = 0.9991). The IC50 for the *E. cuneatum* alkaloid leaf extract could not be obtained because its maximum concentration did not achieve 50% of XO inhibitory activity.

### 3.5 Effect of *E. cuneatum* alkaloid leaf extract on carrageenan-induced paw edema

**Figure 7:**
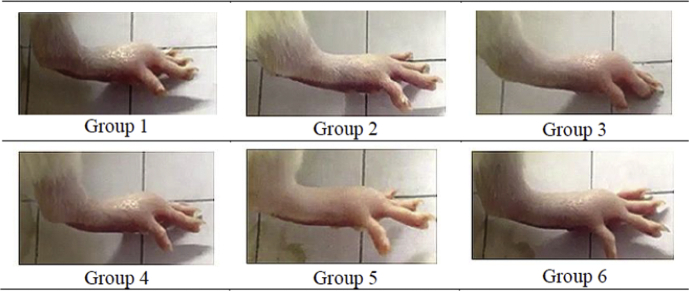
Gross photograph of paw edema of various groups of rats snapped at the 5th hour after the induction of edema. The photograph of each group was the representative of seven rats per group. Group 1 pretreated with 2% Tween 20, Group 2 was pretreated with 300 mg/kg Aspirin, Group 3, 4, 5, and 6 were pretreated with 5 mg/kg, 10 mg/kg, 25 mg/kg, and 50 mg/kg of *E. cuneatum* alkaloid leaf extract respectively.

The gross photographs showed the paw edema thickness of the various pretreated groups of rats at the 5th hour after the induction of edema. Group 2, 5, and 6 showed better effects in reducing the edema upon the carrageenan induction when compared to Group 1 (Figure 7). Group 1 rats pretreated with 2% Tween 20 showed paw edema thickness increased in time-dependent manner upon 1 h of induction, reached its maximum at 4th hour and remained the same at the last hour of monitoring. Group 2 pretreated with 300 mg/kg Aspirin showed significant difference (p < 0.05) of paw edema thickness when compared to Group 1 at the 1st, 2nd, 4th and 5th hour of time interval after induction of edema. Groups 3 and 4 did not show any significant differences (p > 0.05) when compared to Group 1 at all-time intervals. Group 6 showed significant (p < 0.05) anti-inflammatory effect when compared to Group 1 at time interval of the 1st, 4th and 5th hour and Group 5 showed similar pattern as Group 6 except at the 4th hour. The highest percentage of inhibition for Group 2 was at the 5th hour followed by the 1st hour of monitoring and Group 6 showed the highest percentage of inhibition amongst the *E. cuneatum* alkaloid leaf extract pretreated groups (Group 3, 4, and 5) at all the monitored time intervals and manifested a similar pattern as Group 2 (Table 1).

**Table 1:** Effects of different treatments on the increase of paw edema thickness (cm) and the percentage of inhibition that monitored at every time interval (hr).

Increase of paw edema thickness (cm)					
Percentage of inhibition (%)					
	1st hour	2nd hour	3rd hour	4th hour	5th hour
Group 1	0.154 ± 0.005^b,e,f^ (0.00%)	0.229 ± 0.006^b^ (0.00%)	0.270 ± 0.010 (0.00%)	0.283 ± 0.011^b,f^ (0.00%)	0.283 ± 0.011^b,e,f^ (0.00%)
Group 2	0.111 ± 0.003^a,c,d^ (27.92%)	0.188 ± 0.012^a^ (17.90%)	0.233 ± 0.012 (13.70%)	0.219 ± 0.012^a,c^ (22.62%)	0.187 ± 0.011^a,c,d^ (33.92%)
Group 3	0.146 ± 0.006^b,e,f^ (5.19%)	0.217 ± 0.008 (5.24%)	0.254 ± 0.012 (5.93%)	0.274 ± 0.013^b,f^ (3.18%)	0.269 ± 0.010^b,f^ (4.95%)
Group 4	0.151 ± 0.004^b,e,f^ (1.95%)	0.214 ± 0.009 (6.55%)	0.253 ± 0.007 (6.30%)	0.259 ± 0.009^b^ (8.48%)	0.248 ± 0.009^b,f^ (12.37%)
Group 5	0.116 ± 0.004^a,c,d^ (24.68%)	0.200 ± 0.013 (12.66%)	0.250 ± 0.008 (7.41%)	0.243 ± 0.009 (14.13%)	0.229 ± 0.011^a^ (19.08%)
Group 6	0.116 ± 0.002^a,c,d^ (24.68%)	0.197 ± 0.008 (13.97%)	0.236 ± 0.007 (12.59%)	0.221 ± 0.007^a,c^ (21.91%)	0.200 ± 0.008^a,c,d^ (29.33%)

Values expressed as mean ± SEM, n = 7 in each group. Significance values were according to Tukey-HSD post hoc analysis. a is significant when compared to Group 1, negative control group (p < 0.05). b is significant when compared to Group 2, positive control group (p < 0.05). c is significant when compared to Group 3, 5 mg/kg of E. cuneatum (p < 0.05). d is significant when compared to Group 4, 10 mg/kg of E. cuneatum (p < 0.05). e is significant when compared to Group 5, 25 mg/kg of E. cuneatum (p < 0.05). f is significant when compared to Group 6, 50 mg/kg of E. cuneatum (p < 0.05).

### 3.6 Histology results of carrageenan induced acute inflammation in rats

**Figure 8:**
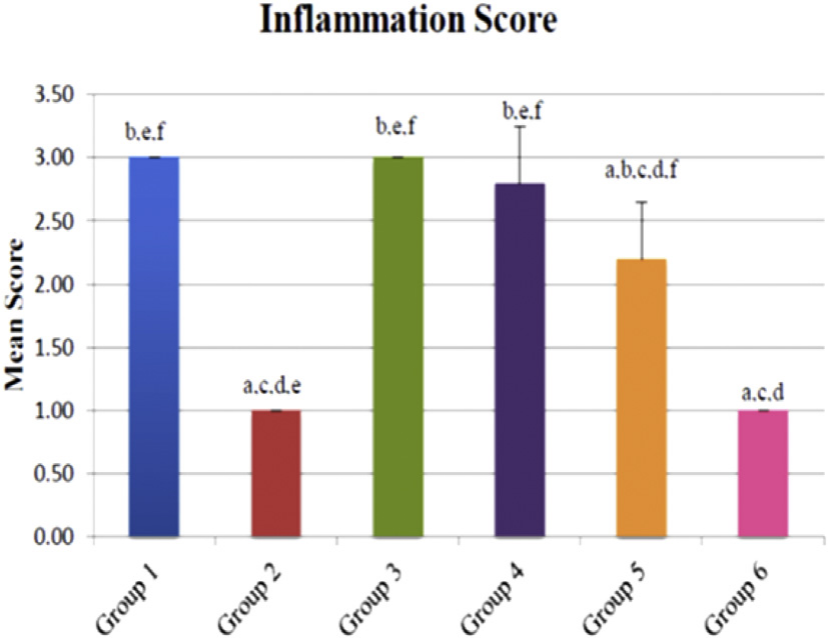
Inflammation score for various treatment groups of rats. Values expressed as mean ± SEM, n = 5 Group 1 pretreated with 2% Tween 20, Group 2 pretreated with 300 mg/kg Aspirin, Group 3, 4, 5, and 6 pretreated with 5 mg/kg, 10 mg/kg, 25 mg/kg, and 50 mg/kg of *E. cuneatum* alkaloid leaf extract respectively. Significance values were according to Tukey-HSD post hoc analysis. a: is significant when compared to Group 1, negative control group (p < 0.05). b: is significant when compared to Group 2, positive control group (p < 0.05). c: is significant when compared to Group 3, 5 mg/kg of *E. cuneatum* (p < 0.05). d: is significant when compared to Group 4, 10 mg/kg of *E. cuneatum* (p < 0.05). e: is significant when compared to Group 5, 25 mg/kg of *E. cuneatum* (p < 0.05). f: is significant when compared to Group 6, 50 mg/kg of *E. cuneatum* (p < 0.05). Key; 0 = no inflammation, 1 = mild inflammation, 2 = moderate inflammation, 3 = severe inflammation.

**Figure 9:**
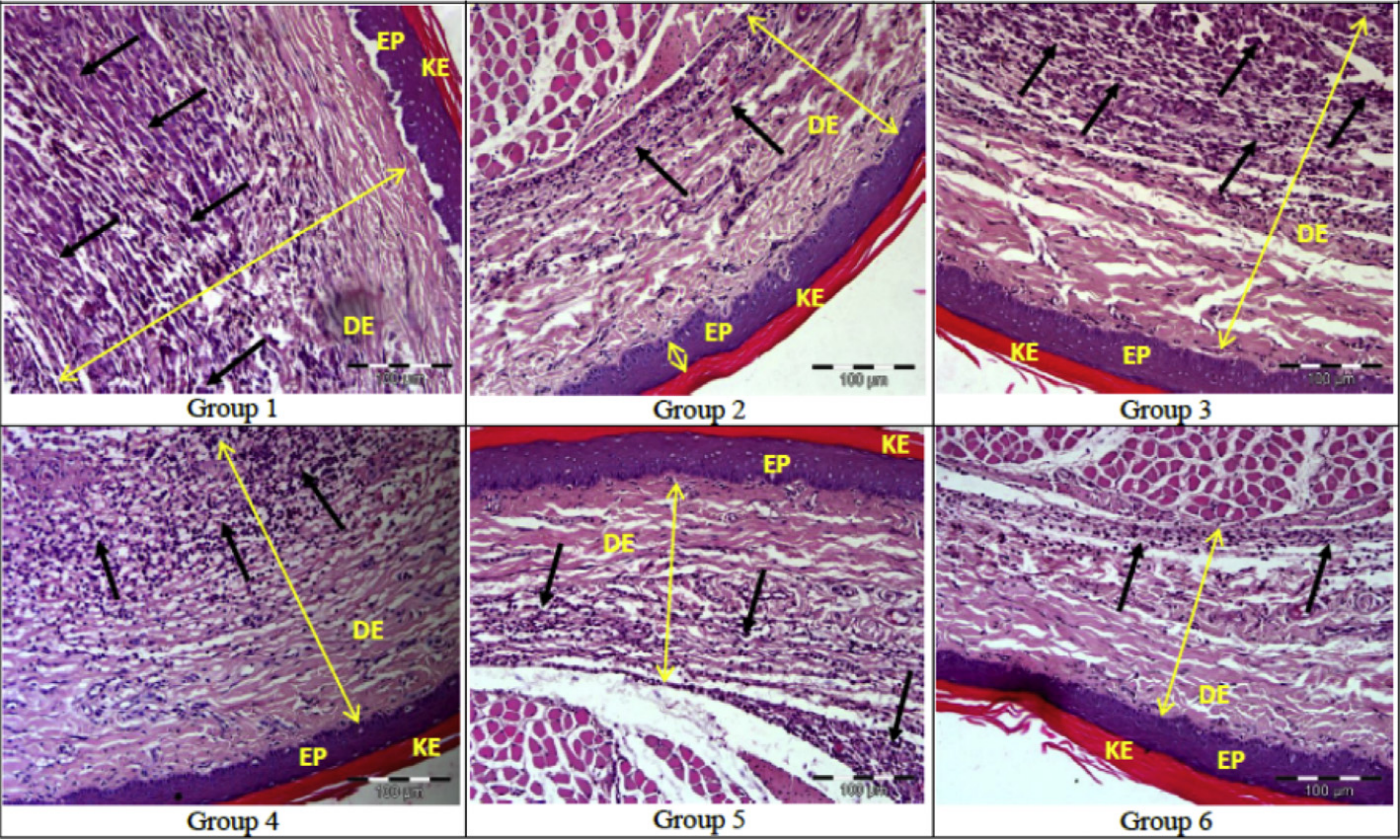
The micrographs of edematous paws of different treatment groups observed under 200x magnification. Black color arrow indicates the leukocyte infiltration to the dermis layer. The leukocytes are displayed as dark purple color. KE is defined as keratin layer, EP is defined as epidermis while DE is defined as dermis. The scale bar was 100 μm for every photograph taken. Group 1 pretreated with 2% Tween 20, Grourp 2 pretreated with 300 mg/kg Aspirin, Group 3, 4, 5 and 6 pretreated with 5 mg/kg, 10 mg/kg, 25 mg/kg and 50 mg/kg of *E. cuneatum* alkaloid leaf extract respectively.

There were significant differences (p < 0.05) between the mean scores of inflammation for Groups 2, 5, and 6 when compared to Group 1, whereas Group 3 and 4 did not displayed any statistically significant differences (p > 0.05) when compared to Group 1. This suggested that, pretreatments administered to Groups 2, 5 and 6 were able to show inhibition of inflammation after induction of edema which is comparable to Group 1. There was no statistical significant differences (p > 0.05) between Group 6 when compared to Group 2 (Figure 8). The micrograph shows that Group 1 demonstrated the most severe form of inflammation with massive infiltration of leukocytes at the dermis layer when compared to the other groups of rats. Edema was extensive in Group 1 with severe disruption and separation collagen tissues seen at the dermis layer. However, Group 2 and 6 showed less massive infiltration of leukocytes as well as less collagen disruption as compared to Group 1, 3, and 4 (Figure 9).

## 4 Discussion

In this study, the percentage yield of the alkaloid extract from *E. cuneatum* leaf powder was 0.12%, which was almost equal to the amount obtained in previous study which reported a yield of 0.08% [18]. El Imam [18] also reported alkaloid acquired from *E. cuneatum* stem and bark were only 0.03%, which is much lower than the yield obtained from leaf. Maria and Angelina [19] reported that the lowest yield of any medicinally useful alkaloid ever produced on a commercial basis was 0.003%.Hence, it can be suggested that the percentage yield of the alkaloid extract obtained from this study is good enough to be used for commercial purpose.

Mayer's reagent test that was performed showed the presence of alkaloid. This was further confirmed with thin layer chromatography results. The TLC plates showed 4 spots of alkaloids in the extract. This was in line with previous study that showed the presence of many alkaloid in *E. cuneatum* leaves extract. As reported by El-Imam [18] and Kanchanapoom [20], tropane alkaloids including (±)-3α,6ß-dibenzoyloxytropane act as principal alkaloid in *E. cuneatum*. Besides, benzoyl- and tigloyl-esters and nicotine were also found in *E. cuneatum* leaves extract. Analogue to the dibenzoyl ester found in *E. cuneatum* is also known as 3a,6b-dicinnamoyloxytropane [18]. These major alkaloids plays an important role in the properties of *E. cuneatum* extract. *E. cuneatum* is also known to have compounds like inamoside 60 -O-L-aarabinofuranoside, (+)-Catechin, quercetin 3-O-alpha-Lrhamnoside, apocynol B, (6S,9R)-roseoside, vomifoliol 9-Oalpha- L-arabinofuranosyl (1- ->6)-beta-D-glucopyranoside inamoside, and citroside A [20]. These alkaloids were all reported without specifically knowing which one exerts the antioxidant & anti-inflammatory properties of *E. cuneatum*.

Preceding studies have revealed that antioxidants worked through various mechanisms of actions, and there is no “universally accepted assay” that can stand alone to quantify the antioxidant capacity of plants extracts, rather multiple assays are being employed [21]. Hence, the present study employed the use of DPPH, FRAP and xanthine oxidase inhibition assay to evaluate the antioxidant capacity of *E. cuneatum* alkaloid leaf extract. In DPPH free radical scavenging assay for this study, the *E. cuneatum* alkaloid leaf extract showed a concentration-dependent relationship which was paralleled to ascorbic acid (the reference standard). This is in conformism to the previous study which reported that the plant extract scavenged the odd electron of the DPPH radical and led to the vanishing of absorption measured at 517 nm in visible spectroscopy. The lower the absorbance measured, the higher the percentage of free radical scavenging activity [22]. Further, this study showed that the EC50 of *E. cuneatum* alkaloid leaf extract (1482 μg/ml) was significantly higher than that of ascorbic acid (4.69 μg/ml). This larger EC50 obtained from *E. cuneatum* alkaloid leaf extract corresponded to its lower antioxidant activity. This findings is in agreement with the previous studies in which the chloroform extract of Cymbopogon citratus and methanolic extract of Swietenia mahagoni displayed lower antioxidant activity when compared to the reference standard, ascorbic acid. The studies suggested the reason behind was due to the low potency of the extract and a higher concentration was required to achieve the maximal scavenging activity as the reference standard. The studies also suggested that although the lower activities were shown by the plant extracts, but it was still evident that the extracts did show some proton-donating activities which could serve as free radical inhibitors or scavengers, acting possibly as primary antioxidants [9, 23].

The present study showed that the FRAP values increased in direct proportion to the concentration of *E. cuneatum* alkaloid leaf extract. This is indicative that the alkaloid extract act as reductone that reduced the ferric tripyridyltriazine [Fe (III)-TPTZ] complex to ferrous tripyridyltriazine [Fe (II)-TPTZ]. This was similar to the previous study which revealed that, the higher the FRAP value the higher the reducing power of the extract on electron transfer ability towards the FRAP reagents [22]. The reference standard used in this study, ascorbic acid displayed significant higher slope in linear curve of FRAP values plotted against various concentrations and smaller EC1 value when compared to the *E. cuneatum* alkaloid leaf extract. Another study reported an existence of positive correlation between total phenolic content from aqueous and methanol extracts with FRAP assay (r2) ranging from 0.954 to 1.00. It was postulated that the positive reducing potential was associated with the presence of phenolic compounds, which exerted their action by breaking the free radical chain through donating a hydrogen atom [24]. This was in agreement to the study conducted by Gutiérrez [25], which explained that the alkaloid extraction of plant will not allow the presence of phenolic compounds in the extract and its antioxidant properties was solely dependent on the alkaloid compounds present in the extract. Previous and current studies proved that the plant *E. cuneatum* did not possess the ability to inhibit XO in the hydroxylation pathway of hypoxanthine to xanthine and xanthine to the production of uric acid. This was justified in which the aforementioned studies were failed to achieve IC50, which is the concentration needed to achieve 50% of XO inhibitory activity. Hence, physiologically it could not inhibit the production of uric acid in the body, therefore it won't be proposed as the alternative treatment for gout arthritis as well as nephrolithiasis [26]. Bala [27], showed that Berberis aetnensis alkaloid extract displayed positive activity in DPPH antioxidant assay and XO inhibition assay at the same time. In contrast, the present study demonstrated positive activity in DPPH antioxidant assay only. The Campisi's studies revealed that the positive activity showed by Berberis aetnensis alkaloid extract was related to its ability to inhibit superoxide anion generation. This is because the process of catalyzing substrate in the purine metabolic pathway involves the production of superoxide anion (O_2_-•) and hydrogen peroxide (H_2_O_2_) when xanthine dehydrogenase was convert to xanthine oxidase [27].

Carrageenan-induced paw edema is one of the primary models used when evaluating new anti-inflammatory drugs, as the model can cause both local and acute inflammatory responses. It is a highly reliable and commonly used model for investigation of paw tissue during acute inflammation [28]. The development of carrageenan-induced paw edema is a biphasic event: the initial phase occurs within one hour upon induction whereas second phase occurs in 2–3 h after the induction [14]. According to Du [29], the initial increase of paw edema thickness was related to the release of serotonin, histamine, thrombin, bradykinin and vascular endothelial growth factor. The further increase of paw edema thickness in the second phase was related to the release of prostaglandins, nitric oxide (NO), oxygen-derived free radicals, as well as local infiltration of neutrophils. In the present study, the rats were pretreated with aspirin or the extracts 1 h before the induction of edema so as to evaluate the prophylactic effects of the *E. cuneatum* alkaloid leaf extract against edema [30]. In the present study, pretreatments of rats with 2% Tween 20 does not show anti-inflammatory activity as continuous increment of paw edema thickness was observed one hour after the induction with carrageenan. Additionally, the results also revealed that 25 mg/kg and 50 mg/kg of *E. cuneatum* alkaloid leaf extract worked similar to 300 mg/kg Aspirin, an established NSAID and a cyclooxygenase (COX-1 and 2) inhibitor, as no statistically significant difference (p > 0.05) of paw edema thickness was observed between the aspirin pretreated group and the *E. cuneatum* pretreated groups of rats. The mechanism of action of aspirin is the same as other NSAIDs, for example indomethacin and celecoxib inhibits cyclooxygenase-induced prostaglandin production and were commonly used as reference drugs in evaluating the anti-inflammatory and analgesic mechanism of plant extracts [31]. As the study done by Maroon [32], revealed that aspirin showed additional action in modulating inflammation reactions. As aspirin worked via inhibition of the NFkß transcription factors in expressing pro-inflammatory factors. Since the *E. cuneatum* alkaloid leaf extract worked similar to aspirin, NF kß could be the possible pathway that the alkaloid extract modulate the inflammation response. Hence, further investigation is needed to explore the exact mechanism of action of the *E. cuneatum* alkaloid leaf extract. The anti-inflammatory activity of *E. cuneatum* leaf extract was studied previously by Saleh [33]. The study used the same experiment model as the present study, but reported that 400 mg/kg of *E. cuneatum* aqueous leaf extract only slowed down the edema progress in rats paw, but did not reduce the edema volume. In contrast to present study, the *E. cuneatum* alkaloid leaf extract showed significant reduction of paw edema when compared to the negative control group. Hence, it is suggestive that the alkaloid chemical constituents could be the main contributors of the anti-inflammatory action of *E. cuneatum* leaf. Aziddin [34], reported that Mitragyna speciosa alkaloid leaf extract showed better anti-inflammatory effects when compared to aqueous and methanol leaf extracts. In addition, the study done by Gutiérrez [22] also showed that Solanum rostratum and Nicotiana trigonophylla alkaloid leaf extracts demonstrated better anti-inflammatory action when compared to their respective methanolic leaf extracts.

In this study, the pretreatment of rats with *E. cuneatum* alkaloid leaf extract led to diminished leukocytes infiltration and collagen tissues disruption in a dose dependent manner. The results also revealed that the effects of the highest dose (50 mg/kg) of the alkaloid extract was similar to that of aspirin. Using different plant extracts, other studies have also demonstrated similar effects. Du [35] reported that essential oil extracted from Cinnamomum longepaniculatum leaf ameliorated the inflammatory cells infiltration and it was similar to the positive control indomethacin which did not affect the connective tissue disruption in carrageenan-induced paw edema model. Further, Fayazuddin [36], also showed that the pretreatment with Moringa oleifera ethanolic extract, showed less extensive edema with mild separation of layers of epidermis, dermis and collagen fibres as well as less infiltration of neutophils that worked similar to aspirin.

The pathogenesis of inflammation involves the excessive activation of phagocytes and generation of free radicals. While the formation of radicals ROS could cause disruption of phospholipids in cellular bilayer and alter its normal function which further worsen the inflammation reaction [28]. Hence, secondary compounds from plants that exhibits antioxidant properties against free radicals formation can stop the ROS generation and limit the oxidative cellular damages. According to previous studies, positive results found in DPPH assay for the plant Vaccinium leschenaultii suggested that it was the free radical scavenging properties portrayed by the extract that was responsible for one of the mechanisms of its anti-inflammatory effects observed in carrageenan-induced paw edema model. Another study done on the plant Osbeckia parvifolia ethanolic extract also displayed the similar results as the aforementioned study and suggested that the positive effect shown in DPPH antioxidant model could contribute some beneficial effects in various inflammation disorders [37, 38]. In summary, plants which exhibits antioxidant property especially in free radical scavenging activity can play a vital role in modulating inflammatory reactions.

## 5 Conclusion

The present study suggest that *E. cuneatum* alkaloid leaf extract is a potential source of antioxidant as it showed an antioxidative properties in DPPH antioxidant assay. The extract also possesses anti-inflammatory properties as it has similar effects to aspirin in carrageenan-induced model of paw edema test. The alkaloids extracts showed a reduction of the edema in a dose dependent manner. This study confirmed the traditional use of this plant for medicinal purposes. However, since the present study do not proceed for further isolation of alkaloids, the exact alkaloids which is exhibiting the major properties of *E. cuneatum* extract need to be further investigated in the future.

## Declarations

### Author contribution statement

M.S. Chiroma: Analyzed and interpreted the data; Wrote the paper.

L.S. Li: Conceived and designed the experiments; Performed the experiments; Wrote the paper.

T. Hashim: Conceived and designed the experiments; Wrote the paper.

S. K. Adam: Performed the experiments; Analyzed and interpreted the data.

Z. Yusuf, M.A.M. Moklas and S.A. Rahman: Contributed reagents, materials, analysis tools or data; Wrote the paper.

### Funding statement

CUCMS Research Grant Scheme (CRGS) (CRGS/03/02/2013) financially supported the study.

### Competing interest statement

The authors declare no conflict of interest.

### Additional information

No additional information is available for this paper.
